# Yoga’s effect on inflammatory biomarkers and metabolic risk factors in a high risk population – a controlled trial in primary care

**DOI:** 10.1186/s12872-015-0086-1

**Published:** 2015-08-19

**Authors:** Moa Wolff, Ashfaque A. Memon, John P. Chalmers, Kristina Sundquist, Patrik Midlöv

**Affiliations:** Center for Primary Health Care Research, Department of Clinical Sciences in Malmö, Lund University, Jan Waldenströms gata 35, Skåne University Hospital, 205 02 Malmö, Sweden; The George Institute for Global Health, Sydney, Australia; Sydney Medical School, University of Sydney, Sydney, Australia; Prevention Research Center, Stanford University School of Medicine, Stanford, CA USA

## Abstract

**Background:**

Yoga can reduce blood pressure and has also been suggested to reduce inflammatory biomarkers and metabolic risk factors for cardiovascular diseases (CVDs). We aimed to assess the benefit of two yoga interventions on inflammatory biomarkers and metabolic risk factors in a high risk population in primary care.

**Methods:**

Adult patients from a health care center in Sweden, with diagnosed hypertension, were invited to undergo a baseline check at the health care center. Baseline check included standardized blood pressure measurement, BMI and weight circumference measurements, blood sampling (hs-CRP, IL-6, FP-glucose, HbA1c, cholesterol, TG, LDL and HDL) and a questionnaire on self-rated quality of life (WHOQOL-BREF). There were three groups: 1) yoga class with yoga instructor; 2) yoga at home; and 3) a control group. In total, 83 patients were included and matched at the group level for systolic blood pressure. A majority of the patients (92 %) were on antihypertensive medication, which they were requested not to change during the study. After 12 weeks of intervention, the assessments were performed again.

**Results:**

We recorded no evidence that yoga altered inflammatory biomarkers or metabolic risk factors in our study population. A total of 49 participants (59 %) met the criteria for metabolic syndrome.

**Conclusion:**

The yoga interventions performed in our study did not affect inflammatory biomarkers or metabolic risk factors associated with CVD in the study population of primary care patients with hypertension. Further randomized trials are needed to elucidate the effects of yoga on CVD risk factors in this particular group.

**Trail registration:**

NCT01302535, February 22, 2011.

## Background

Yoga has been shown to reduce blood pressure (BP) in several studies [1, 2]. In the YHIP study (Yoga’s effect on Hypertension In Primary care) of yoga in primary health care patients with hypertension, we showed that a short home-based yoga program had an antihypertensive effect and improved self-rated quality of life [3].

Persistent hypertension increases the risk of developing coronary heart disease, stroke and other cardiovascular diseases (CVDs), such as heart failure [4].

The metabolic biomarkers HbA1c, fasting plasma (FP-) glucose, cholesterol, triglycerides (TGs) and low density lipoproteins (LDL) are known risk factors for CVDs and they are often associated with elevated blood pressure as part of the metabolic syndrome [5, 6]. The opposite applies to high density lipoproteins (HDL), where high levels seem to protect against CVD [7]. The metabolic syndrome is a combination of certain risk factors that multiply the risk for heart disease, diabetes and stroke [8]. To be diagnosed with metabolic syndrome, a patient has to have at least three of the following five conditions: Central obesity (waist circumference ≥ 102 cm or ≥ 88 cm in male and female respectively); Blood pressure ≥ 130/85 mmHg (or receiving drug therapy for hypertension); Triglycerides ≥ 1.7 mmol/L (or receiving therapy for hypertriglyceridemia); HDL < 1.0 mmol/L or < 1.3 mmol/L in male and female respectively) and; FP-glucose ≥5.6 mmol/L (or receiving drug therapy for hyperglycemia) [9]. Other studies have shown that dietary intervention combined with yoga has a positive effect on these metabolic biomarkers [9, 10].

The inflammatory blood tests high-sensitive C-reactive protein (hs-CRP), interleukin 6 (IL-6) and interleukin 10 (IL-10) reflect the degree of inflammation in the body, and high levels of hs-CRP (≥2.78 mg/L) and IL-6 (≥3.19 pg/mL) have been associated with increased risk of death [11]. High levels of hs-CRP also seem to be a predictor of cardiovascular events [12]. IL-10 is a cytokine with anti-inflammatory effects. Some studies suggest that yoga practice lowers the levels of hs-CRP and IL-6 [12, 13] and increases levels of anti-inflammatory proteins that in turn increases IL-10 levels [14]. Previous studies also indicate a connection between hypertension alone and systemic low-grade inflammation [15–17].

The effects of yoga on inflammatory biomarkers and metabolic risk factors have, however, not been studied in a primary care setting, although most patients with hypertension and metabolic syndrome are treated in primary care. The aim of the study was to assess the benefit of yoga on inflammatory biomarkers and metabolic risk factors in a high risk population of primary health care patients with hypertension.

## Methods

### Design

The YHIP study, from which the blood samples are taken, was a prospective three-arm single-center study of the effects of two types of yoga on BP and quality of life [3]. There were two intervention groups and one control group. The study was designed as a matched controlled open clinical trial. Blood tests, BMI-, waist circumference-, BP measurements and assessments of lifestyle, health status and quality of life [18] were carried out at baseline and after 12 weeks of intervention. The groups were matched based on SBP after baseline assessments.

### Patients and recruitment

In January 2011, adult patients with diagnosed hypertension were identified by electronic charts search at Svedala Health Care Center in Southern Sweden. Patients were invited to participate if their BP when most recently measured at the health care center was between 120 and 160/80 and 100 mm Hg (e.g. normal, high normal and grade 1 hypertension levels). The patients who agreed to participate were invited to the health care center for baseline assessments. Patients with BP values of 120–179/<110 mmHg in the baseline check were eligible for enrollment. Fasting blood samples were collected and analyzed for HbA1c, FP-glucose, cholesterol, TGs, HDL, LDL and hs-CRP. For each patient three cryo tubes with blood were frozen for future analysis of IL-6 and IL-10. Baseline assessments and study questionnaires (lifestyle and health status survey and quality of life were completed after written informed consent was obtained from the participants.

All participants were requested not to change their medication during the study, and any change in medication was registered at follow-up after 12 weeks. For further details regarding patients and procedures please refer to our previously published article [3].

### Interventions

The yoga practiced in the YHIP study is a form of Kundalini yoga developed at the Institute for Medical Yoga (IMY) in Stockholm, Sweden [19]. Intervention group 1 (28 persons) was divided into three smaller groups, each consisting of 8–12 participants. Each group met once a week for 60 min at the health care center to practice yoga with a yoga instructor. The participants were encouraged to practice yoga 30 min every day at home between the yoga classes. The participants in intervention group 2 (28 persons) were each given a doctor’s appointment (20 min) during which they received instructions for two yoga exercises to perform at home for a combined total of 15 min a day. No changes were made for the participants in the control group, who received treatment as usual. In order to evaluate compliance with yoga practice, each participant received a yoga calendar in which to record when they did yoga. For further details regarding the interventions please refer to our previously published article [3].

### Outcome

The main outcome measure was change in blood inflammatory and metabolic factor levels. We chose to analyze inflammatory and metabolic factors with known or suspected connections to CVD.

### Measurements

Blood samples were collected at baseline and follow-up for assessment of the following factors: HbA1c, FP-glucose, cholesterol, TGs, HDL, LDL and hs-CRP. The blood samples were drawn in the morning after a fast since midnight.

HbA1c was analyzed using the Bio-Rad Variant II chromatographic method (reference range 31–44 mmol/mol) by Swedish Mono-S high-performance ion-exchange liquid chromatography. Due to an instrument change at the laboratory during the intervention period, the following were analyzed by different methods at baseline and follow-up: FP-glucose, cholesterol, TGs and HDL. At baseline, they were measured in plasma using an LX20 analyzer (Beckman Coulter Inc., Brea, CA). At follow-up the above samples were analyzed using a cobas 6000 Analyzer (Roche Diagnostics, SA). LDL level was calculated using the Friedewald formula [20]. The results from follow-up were recalculated according to regression equations provided by the laboratory to compensate for any differences due to the change in instrument.

The blood samples from baseline and follow-up were used for IL-6 and IL-10 levels. Serum was isolated from blood by centrifugation at 10,000 g at 4 °C for 10 min. Samples were analyzed using Bio-Plex Pro human cytokines assay (Bio-Rad Inc., Hercules, CA), according to the manufacturer’s instructions with a few modifications. Briefly, samples were diluted 1:3 in the sample diluent provided with the kit and incubated with magnetic beads coupled to specific antibodies. IL-6 and IL-10 were detected with premixed detection antibody. Beads were read on a Bio-Plex Suspension Array System and the data were analyzed using Bio-Plex Manager™ software (version 4.0). Absolute concentrations were measured from a standard curve generated from nine serially diluted standards provided with the kit. Each sample was analyzed in duplicate. Values are presented in pg/mL. Each run included controls of known concentration for each cytokine and a blank.

The health status and lifestyle survey was designed for this study and is not validated. The survey contains questions regarding comorbidity for diabetes and cardiovascular disease, smoking and drinking habits and physical activity.

The participants were instructed to mark the dates they completed the yoga training. At follow-up, they submitted their yoga calendars. The information in the calendars was not controlled or questioned.

BP was measured in a standardized way, in a sitting position after 5–10 min of rest with validated electronic BP devices. The mean of two readings were calculated (mean of three when the first and second reading differed by >5 mm Hg). The measurements were carried out by trained nurses and care assistants.

### Statistical analysis

Assuming a mean treatment difference in SBP of 5 mmHg between the yoga at home and control groups, a standard deviation of 6 mmHg and a drop-out rate of 30 %, 33 patients per group would provide 80 % power to detect a statistically significant difference at the 5 % level using a two-sided test.

One-way-ANOVA was used to determine whether there were any significant differences in baseline data between the groups. For the laboratory results that were not normally distributed (FP-glucose, HbA1c, hs-CRP, IL-10 and IL-6) we used the Kruskal-Wallis test instead.

Differences in blood test parameters, BP and waist circumference between baseline and follow-up in each group were calculated by paired-samples Student’s *t*-test. Differences in mean change between the yoga groups and the control group were calculated by independent-samples Student’s *t*-test. For the laboratory results that were not normally distributed (FP-glucose, HbA1c, hs-CRP, IL-6 and IL-10), the differences within and between groups were calculated by Wilcoxon test and Mann–Whitney *U*-test, respectively.

Version 22 of the IBM SPSS Statistics was used for the statistical analysis.

All patients who attended follow-up appointments were included in the analyses (as observed cases, OC). We also made calculations in which patients who did not perform yoga in 9/12 weeks or who changed their medication were excluded (per-protocol set, PPS). This criterion (9/12 weeks) was set up together with the IMY founder, and it was not known to the patients.

### Ethical aspects

The study conforms to the principles outlined in the Declaration of Helsinki and was approved by the Regional Ethical Review Board in Lund, Sweden (2010/728). The study was registered at ClinicalTrials.gov (NCT01302535).

## Results

**Fig. 1:**
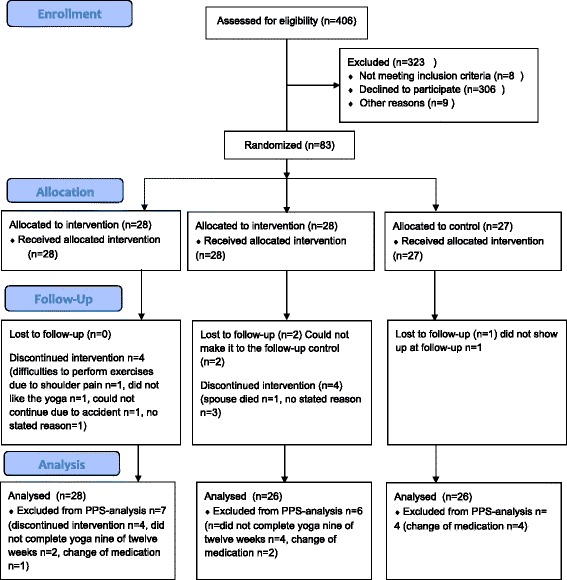
CONSORT 2010 flow diagram

Figure 1 shows the flow of participants through the study. Of the 406 patients that where invited, 75 % (306) declined to participate. At the baseline assessment 8 patients did not meet the inclusion criteria regarding blood pressure. Only three participants where lost to follow-up. The baseline characteristics of the patients are presented in Table 1. There was a predominance of women in all three groups. A majority of the patients (72 %) were overweight (body mass index (BMI) > 25 kg/m^2^) and 60 % fulfilled the criteria for metabolic syndrome (Table 1). All participants were diagnosed with hypertension at enrollment, and 92 % were on hypertensive medication (Table 1).

**Table 1:** Baseline characteristics

	Intervention group 1	Intervention group 2	Group 3
	Yoga class group	Yoga at home group	Control group
	*n* = 28	*n* = 28	*n* = 27
	Mean (SD)^a^	Mean (SD)^a^	Mean (SD)^a^
Age (years)	66.2 (7.7)	64.0 (10.3)	60.8 (11.0)
Female, number (%)	19 (67.9)	20 (71.4)	16 (59.3)
Metabolic syndrome, number (%)	19 (67.9)	13 (48.1)	17 (63.0)
BMI (kg/m^2^)	29.7 (7.0)	28.5 (7.3)	28.8 (4.0)
Waist circumference, (cm)	100.4 (14.5)	97.0 (15.2)	100.9 (9.9)
Cholesterol (mmol/L)	5.2 (1.0)	5.4 (1.1)	5.3 (1.2)
LDL (mmol/L)	3.3 (0.9)	3.5 (1.0)	3.4 (1.1)
HDL (mmol/L)	1.3 (0.4)	1.5 (0.4)	1.4 (0.4)
TGs (mmol/L)	1.3 (1.0)	1.2 (0.8)	1.5 (1.2)
FP-glucose (mmol/L)	5.5 (0.8)	5.4 (1.6)	5.9 (2.3)
HbA1c (mmol/mol)	40.9 (10.5)	40.0 (8.5)	39.6 (10.1)
hs-CRP (mg/L)	5.0 (3.6)	2.9 (2.2)	3.6 (3.6)
IL-6 (pg/mL)	5.4 (3.3)	6.6 (5.1)	5.2 (3.6)
SBP (mmHg)	143.8 (14.9)	143.6 (14.2)	144.3 (14.5)
DBP (mmHg)	89.0 (7.6)	88.4 (6.2)	89.8 (7.3)

^a^Unless otherwise indicated

*SBP* Systolic blood pressure, *DBP* Diastolic blood pressure, *LDL* Low-density lipoprotein, *HDL* High-density lipoprotein, *TGs* Triglycerides, *hs-CRP* High-sensitivity C-reactive protein, *IL-6* Interleukin 6

Table 2 shows changes in blood parameters, systolic BP (SBP) and diastolic BP (DBP) for the three groups. No significant differences in change in SBP from baseline between the yoga groups and the control group were detected. However, the improvement in DBP for the yoga at home group was significantly greater than that for the control group. The increase in TG level was significantly higher in the yoga class group compared to the control group (+0.3 ± 0.6 vs. -0.3 ± 1.1 mmol/L). No significant between-group differences in change from baseline were detected for any of the other metabolic or inflammatory blood factors or for waist circumference. IL-10 was not detectable in a majority of the patients (59 % at baseline and 63 % at follow up), and we therefore chose not to present the results.

**Table 2:** Change from baseline and difference vs. control for blood parameters, waist circumference, SBP and DBP

	Intervention group 1		Intervention group 2		Group 3	
	Yoga class group		Yoga at home group		Control group	
	OC	PPS	OC	PPS	OC	PPS
	*n* = 28	*n* = 21	*n* = 26	*n* = 20	*n* = 26	*n* = 22
	Mean (CI)	Mean (CI)	Mean (CI)	Mean (CI)	Mean (CI)	Mean (CI)
Cholesterol (mmol/L) change from baseline	0.3 (0.1–0.5)*	0.2 (−0.0–0.4)	−0.1 (−0.3–0.2)	−0.1 (−0.2–0.4)	0.0 (−0.3–0.3)	0.1 (−0.3–0.4)
Difference vs. control	0.3 (−0.1–0.6)	0.1 (−0.2–0.5)	−0.1 (−0.4–0.3)	−0.1 (−0.4–0.3)		
LDL (mmol/L) change from baseline	0.1 (−0.0–0.3)	0.1 (−0.1–0.3)	−0.2 (−0.5–0.1)	−0.1 (−0.3–0.2)	0.0 (−0.3–0.3)	0.0 (−0.3–0.3)
Difference vs. control	0.2 (−0.2–0.5)	0.1 (−0.3–0.5)	−0.2 (−0.6–0.2)	−0.1 (−0.5–0.4)		
HDL (mmol/L) change from baseline	0.0 (−0.1–0.1)	0.0 (−0.1–0.1)	0.0 (−0.1–0.1)	0.0 (−0.0–0.1)	0.0 (−0.1–0.1)	0.0 (−0.1–0.1)
Difference vs. control	−0.0 (−0.1–0.1)	−0.0 (−0.2–0.1)	−0.0 (−0.1–0.1)	−0.0 (−0.1–0.1)		
TGs (mmol/L) change from baseline	0.3 (0.1–0.5)*	0.3 (0.0–0.5)*	0.1 (−0.1–0.2)	0.1 (−0.1–0.2)	−0.3 (−0.8–0.1)	−0.3 (−0.8–0.2)
Difference vs. control	0.6 (0.1–1.1)*	0.6 (0.0–1.1)*	0.4 (−0.1–0.8)	0.4 (−0.1–0.9)		
FP-glucose (mmol/L) change from baseline	0.1 (−0.2–0.4)	0.0 (−0.3–0.3)	−0.1 (−0.3–0.2)	−0.1 (−0.4–0.2)	−0.4 (−1.1–0.3)	−0.4 (−1.2–0.4)
Difference vs. control	0.5 (−0.2–1.3)	0.4 (−0.5–1.3)	0.4 (−0.4–1.1)	0.3 (−0.6–1.2)		
HbA1c (mmol/mol) change from baseline	−1.4 (−3.9–1.2)	−1.9 (−4.9–1.1)	−0.2 (−1.5–1.1)	0.3 (−1.3–1.9)	−0.8 (−2.0–0.3)	−0.9 (−2.2–0.4)
Difference vs. control	−0.5 (−3.3–2.3)	−1.0 (−4.0–2.0)	0.6 (−1.1–2.3)	1.2 (−0.7–3.2)		
hs-CRP (mg/L) change from baseline	−0.8 (−2.2–0.5)	−0.6 (−2.3–1.1)	0.2 (−0.6–1.0)	0.1 (−1.0–1.2)	0.4 (−1.2–2.1)	0.5 (−1.4–2.4)
Difference vs. control	−1.2 (−3.3–0.8)	−1.1 (−3.6–1.3)	−0.2 (−2.0–1.6)	−0.4 (−2.7–1.8)		
IL-6 (pg/mL) change from baseline	−0.8 (−2.3–0.7)	−0.8 (−2.4–0.9)	1.1 (−0.2–2.4)	1.1 (−0.4–2.5)	1.0 (−0.8–2.8)	1.2 (−0.7–3.1)
Difference vs. control	−1.8 (−4.1–0.5)	−2.0 (−4.5–0.5)	0.2 (−1.9–2.3)	−0.1 (−2.5–2.2)		
Waist circumf. (cm) change from baseline	−0.4 (−1.9–1.1)	−0.1 (−1.9–1.6)	0.6 (−1.5–2.7)	0.8 (−1.9–3.5)	−0.0 (−2.3–2.3)	0.1 (−2.4–2.6)
Difference vs. control	−0.4 (−3.1–2.2)	−0.2 (−3.2–2.8)	0.6 (−2.4–3.7)	0.7 (−2.8–4.3)		
SBP (mmHg)^a^ change from baseline	0.3 (−5.8–6.4)	−0.2 (−8.1–7.8)	−6.8 (−11.6–2.11)*	−6.1 (−11.7–0.7)	−2.3 (−7.6–3.0)	−1.9 (−7.7–4.1)
Difference vs. control	2.6 (−5.4–10.5)	1.7 (−7.9–11.1)	−4.4 (−11.5–2.4)	−4.2 (−12.3–3.5)		
DBP (mmHg)^a^ change from baseline	0.2 (−3.2–3.7)	0.3 (−3.9–4.3)	−4.4 (−7.3–1.3)*	−3.9 (−7.3–0.1)	0.8 (−3.2–4.4)	1.0 (−3.2–4.9)
Difference vs. control	−0.6 (−5.3–4.6)	−0.8 (−6.2–5.0)	−5.2 (−9.6–0.2)*	−4.9 (−9.9–0.1)*		

*p < 0.05 vs. control group

^a^previously published results [3]

*C*I Confidence interval, *DBP* Diastolic blood pressure, *HDL* High-density lipoprotein, *hs-CRP* High-sensitivity C-reactive protein, *LDL* Low-density lipoprotein, *OC* Observed cases, *PPS* Per-protocol set, *SBP* Systolic blood pressure, *SE* Standard error of the mean, *TGs* Triglycerides

The PPS consists of all patients who practiced yoga at least once a week for 9 weeks or more and who had no change in medication during the study period

Compliance with yoga practice (number of yoga sessions) was lower in the yoga class group than in the yoga at home group. However, the average total time spent on yoga practice was higher in the yoga class group than in the yoga at home group (about 24 h vs. 16 h).

We also did separate analyzes for those patients whose BP decreased during the study (group one and two), without finding any significant change in any of the blood test before and after intervention (data not shown).

## Discussion

We recorded no evidence that yoga altered inflammatory or metabolic biomarkers in our high risk population of primary health care patients with hypertension. Although previous studies have shown that yoga can reduce BP, our study could not detect any effects on the other metabolic risk factors examined. The effects of yoga on inflammatory biomarkers and metabolic risk factors have not been studied previously in this particular group, although most patients with hypertension and metabolic syndrome are treated in primary health care and although there is increasing interest in and uptake of yoga at community level.

Previous studies have shown that yoga practice can reduce levels of inflammatory factors such as hs-CRP and IL-6 [21, 22]. However, none of these studies focused on patients with hypertension or metabolic syndrome, but on patients with chronic heart failure and breast cancer respectively. Nor did they look at yoga’s effect on BP and metabolic risk factors, but on exercise capacity, mood and fatigue.

Other studies have looked at yoga’s effect on metabolic parameters such as glucose and lipid levels in patients with CVDs [8, 9, 22]. In these studies, in contrast to our study, the yoga interventions were combined with other measures such as changes in diet. Two out of the three studies showed effects on the metabolic parameters [8, 9].

One explanation for the lack of significant results of our study could be that the patients have already influenced their biomarker levels through their medication (BP-lowering, anti-inflammatory aspirin and lipid-lowering statins) to the extent that yoga has no additional affect. There are studies suggesting that antihypertensive treatment alone attenuates circulating levels of IL-6 [23]. Since 92 % of the study participants were already on antihypertensive medication at baseline, the possibility of further reducing IL- 6 levels could thus have been adversely affected.

Another reason for the lack of effect is that there are many different yoga schools, and we can’t exclude the possibility that certain types of yoga have greater impact on inflammatory and metabolic blood factors. The yoga intervention design in the above described studies varied and the length of the intervention ranged from 8 to 12 weeks, making it difficult to compare the interventions in terms of effectiveness. However, the findings could also mean that yoga cannot contribute to improve the metabolic profile or decrease inflammation in these patients.

The study has a number of strengths. Primarily, it is more comprehensive compared to other similar yoga studies.

It also investigated the effect of yoga on a large number of biomarkers and risk factors. The study focused on a high risk population, where there is often a need for additional treatment to achieve treatment goals. Strengths of this study also include excellent adherence with minimal attrition: only 3 of 83 patients failed to attend the follow-up.

On the other hand the study has a number of limitations. Our study is limited to a single form of yoga [19]. It may be that other schools of yoga or other yoga programs have a better impact on the biomarkers and risk factors that we studied. The participants were matched at a group level and not randomized. However, since there was no change in blood factor levels in any of the groups throughout the study, it is unlikely that randomization would have made a difference to the outcome in this case. Our rationale for matching the groups was that we wanted to ensure similar SBP values at baseline. A weakness of the study also concerns the self-reported data (yoga calendar), which is a problem in all studies of this kind. Due to an instrument change at the laboratory during the intervention period, four out of nine blood samples were analyzed by different instruments at baseline and follow up. Even though we had regression equations to compensate for the differences, we cannot exclude the possibility that the instrument change influenced the results of these tests to some extent.

For yoga to be considered a viable and accepted treatment alternative for patients with high BP and increased risk of CVD, we need research that can not only show yoga’s effects on BP and on CVD related risk factors, but also help us understand its mechanism of action.

## Conclusion

Although yoga can lower BP and increase quality of life, our study, which is the first in this particular group of patients, could not confirm that the yoga performed had any effect on the biomarkers examined. Further research is needed to confirm the antihypertensive effect of yoga and to clarify how yoga affects BP and other risk factors for CVDs in hypertensive patients in a primary health care setting.

## Abbreviations

BMI: Body mass index
BP: Blood pressure
CVDs: Cardiovascular diseases
DBP: Diastolic BP
FP-glucose: Fasting plasma glucose
HDL: High density lipoproteins
hs-CRP: High sensitive C-reactive protein
IL-6: Interleukin 6
IL-10: Interleukin 10
IMY: Institute for Medical Yoga
LDL: Low density lipoproteins
OC: Observed cases
PPS: Per-protocol set
RAAS: Renin angiotensin aldosterone system
SBP: Systolic BP
TGs: Triglycerides
